# Navigating the Future: The Transformative Impact of Artificial Intelligence on Hospital Management- A Comprehensive Review

**DOI:** 10.7759/cureus.54518

**Published:** 2024-02-20

**Authors:** Shefali V Bhagat, Deepika Kanyal

**Affiliations:** 1 Hospital Administration, Jawaharlal Nehru Medical College, Datta Meghe Institute of Higher Education and Research, Wardha, IND

**Keywords:** interoperability, ethical considerations, data privacy, healthcare innovation, hospital management, artificial intelligence

## Abstract

This comprehensive review explores the transformative impact of artificial intelligence (AI) on hospital management, delving into its applications, challenges, and future trends. Integrating AI in administrative functions, clinical operations, and patient engagement holds significant promise for enhancing efficiency, optimizing resource allocation, and revolutionizing patient care. However, this evolution is accompanied by ethical, legal, and operational considerations that necessitate careful navigation. The review underscores key findings, emphasizing the implications for the future of hospital management. It calls for a proactive approach, urging stakeholders to invest in education, prioritize ethical guidelines, foster collaboration, advocate for thoughtful regulation, and embrace a culture of innovation. The healthcare industry can successfully navigate this transformative era through collective action, ensuring that AI contributes to more effective, accessible, and patient-centered healthcare delivery.

## Introduction and background

The healthcare industry has recently witnessed a significant paradigm shift by integrating cutting-edge technologies. One of the most transformative forces at play is Artificial Intelligence (AI). As hospitals and healthcare organizations grapple with complex challenges, ranging from the efficient management of resources to the delivery of high-quality patient care, AI emerges as a promising solution with the potential to revolutionize the landscape of hospital management [1]. The background of this transformative shift lies in the exponential growth of healthcare data, advancements in computing power, and breakthroughs in AI algorithms. The convergence of these factors has opened new avenues for enhancing hospital management practices' efficiency, accuracy, and overall effectiveness [2].

The significance of AI in hospital management extends beyond mere technological innovation; it represents a fundamental reshaping of how healthcare institutions operate. AI can optimize numerous facets of hospital management, including administrative processes, clinical decision-making, and patient engagement. By leveraging machine learning, natural language processing, and other AI technologies, hospitals can streamline operations, improve patient outcomes, and ultimately redefine the standard of care [3]. Moreover, the significance of AI in hospital management is underscored by its potential to address longstanding challenges faced by the healthcare industry, such as resource constraints, rising costs, and the increasing demand for personalized and efficient healthcare services. The transformative impact of AI holds the promise of enhancing the capabilities of healthcare professionals and revolutionizing the entire patient-care continuum [4].

This comprehensive review aims to delve into the multifaceted impact of AI on hospital management, providing a thorough exploration of its applications, challenges, and future implications. By synthesizing current knowledge and emerging trends, this review aims to serve as a valuable resource for healthcare professionals, administrators, policymakers, and researchers seeking a nuanced understanding of AI's role in shaping the future of healthcare delivery. Through a systematic examination of existing literature, case studies, and real-world implementations, this review will offer insights into how AI reshapes administrative functions, clinical operations, and patient experiences within hospital settings. Additionally, it will address the ethical, legal, and practical considerations associated with integrating AI, providing a holistic view of the opportunities and challenges that lie ahead in navigating the future of hospital management.

## Review

Applications of AI in hospital management

Administrative Functions

Data management: The application of Artificial Intelligence (AI) in data management revolutionizes handling extensive healthcare information within hospital administration. AI algorithms, adept at organizing and analyzing Electronic Health Records (EHRs), ensure rapid access to pertinent patient data. This heightens the precision of administrative decision-making and contributes to elevated patient care standards by facilitating timely and well-informed interventions. The integration of AI in data management addresses the challenge of information overload, allowing healthcare organizations to glean actionable insights from vast datasets [5].

Workflow optimization: AI is pivotal in enhancing administrative workflows, effectively minimizing inefficiencies, and optimizing overall operational performance. Through process automation and intelligent scheduling, AI streamlines staff allocation, appointment scheduling, and routine tasks. This results in a marked increase in productivity, affording healthcare professionals more time to dedicate to direct patient care. The consequential improvement in the efficiency of healthcare services benefits healthcare providers and translates to higher quality and quicker delivery of care to patients [6].

Resource allocation: Addressing the perennial challenge of efficient resource allocation in hospital management, AI-driven predictive analytics emerges as a transformative solution. These analytics optimize resource allocation across various domains, such as staffing levels, medical supplies, and facility utilization by scrutinizing historical data, current trends, and future projections. Through informed and data-driven decision-making, AI contributes to cost savings, enhances resource utilization, and fosters a healthcare system more responsive to the dynamic demands of patient care. The predictive nature of AI in resource allocation ensures that hospitals can proactively adjust their operations, minimizing waste and maximizing the impact of available resources [7].

Clinical Operations

Diagnostics and imaging: The integration of AI applications in diagnostics and medical imaging represents a groundbreaking advancement in healthcare. Machine learning algorithms, trained on extensive datasets, demonstrate remarkable proficiency in analyzing medical images, including X-rays, MRIs, and CT scans. By detecting nuanced anomalies that may escape human observation, AI aids healthcare professionals in achieving swifter and more precise diagnoses. This transformative technology accelerates the diagnostic timeline and significantly enhances treatment planning, optimising healthcare services' efficiency [8].

Treatment planning: AI plays a pivotal role in developing personalized treatment plans by leveraging advanced analytics on patient data, medical histories, and treatment outcomes. The integration of clinical decision support systems powered by AI empowers healthcare professionals to tailor treatment approaches based on the unique characteristics of each patient. This personalized approach improves treatment efficacy and minimizes adverse effects, contributing to a more patient-centric and precise healthcare experience. By synthesizing a wealth of patient information, AI enhances decision-making, ensuring that treatment plans are effective and aligned with individual patient needs [9].

Predictive analytics for patient outcomes: The predictive analytics capabilities of AI represent a paradigm shift in proactive patient care. Through analyzing historical data and real-time patient information, AI algorithms can forecast potential health complications, providing healthcare providers with the foresight to intervene early and prevent adverse events. This proactive approach improves patient outcomes and mitigates the overall burden on healthcare resources. By leveraging predictive analytics, healthcare professionals can implement timely interventions, enhancing the quality of care and optimizing the utilization of resources within the healthcare system [9].

Patient Engagement and Experience

Personalized healthcare: AI emerges as a catalyst for ushering in a new era of personalized healthcare experiences by delving into patient data, preferences, and historical interactions. Through sophisticated analysis, AI tailors treatment plans, generates medication reminders and crafts health education materials uniquely suited to individual needs. This approach cultivates a more engaged and empowered patient population, fostering heightened satisfaction and adherence to treatment plans. By embracing personalization through AI, healthcare providers can forge stronger patient connections, improving long-term health outcomes and a more holistic approach to individual well-being [10].

Virtual health assistants: The integration of AI-powered virtual health assistants, equipped with natural language processing capabilities, transforms the patient experience by providing instantaneous access to medical information, facilitating appointment scheduling, and offering fundamental healthcare guidance. These virtual assistants serve as a bridge for communication between patients and healthcare providers and offer a convenient and accessible means of support. The seamless interaction these virtual assistants enable improves patient engagement and ensures that individuals have readily available resources to navigate their healthcare journey effectively [11].

Remote monitoring: AI-enabled remote monitoring solutions represent a paradigm shift in patient care, empowering individuals to manage their health conditions from their homes actively. By continuously collecting and analyzing data from wearables and other remote monitoring devices, AI identifies potential health issues and promptly alerts healthcare providers in real-time. This proactive approach to healthcare enhances patient outcomes by enabling early intervention and reduces the need for frequent hospital visits. Integrating AI in remote monitoring improves healthcare delivery efficiency and aligns with the growing emphasis on patient-centric care and the broader trend of shifting healthcare beyond traditional hospital settings [12].

AI-driven decision support systems

Clinical Decision Support

Clinical decision support systems (CDSS) driven by artificial intelligence (AI) are rapidly becoming indispensable tools for healthcare professionals, significantly aiding in the intricate landscape of clinical decision-making. These systems represent a pivotal convergence of advanced technologies and vast healthcare knowledge, utilizing extensive patient data, comprehensive medical literature, and real-time information to offer evidence-based recommendations to clinicians [13]. The strength of AI algorithms within these systems lies in their ability to assist in diagnosing medical conditions, propose tailored treatment plans, and predict potential outcomes based on a multifaceted analysis of diverse datasets. By seamlessly integrating data analytics and machine learning, these AI-powered systems augment the precision and efficiency of clinical decision-making processes. Consequently, healthcare professionals benefit from more accurate diagnoses, optimized treatment strategies, and the ability to foresee potential patient outcomes. The overall impact of AI-driven clinical decision support systems extends to enhancing patient care, marking a transformative shift in the healthcare landscape towards more informed, efficient, and patient-centric practices [14].

Operational Decision Support

Beyond their impact on clinical decision-making, AI-driven decision support systems are invaluable tools for optimizing the operational facets of hospital management. These systems harness the power of AI to analyze extensive datasets associated with resource utilization, staff scheduling, and workflow efficiency. By discerning patterns and trends within these datasets, AI assists hospital administrators in making informed decisions that result in enhanced operational effectiveness [15]. This extends to the optimization of inventory management, the prediction of patient admission rates, and an overall improvement in the efficiency of healthcare delivery. Implementing operational decision support systems contributes significantly to cost savings, ensuring judicious resource allocation and fostering a more responsive healthcare infrastructure. Through the synergistic application of AI in operational decision-making, hospitals can achieve financial efficiencies and elevate patient service quality and responsiveness [16].

Ethical Considerations in AI-driven Decision-Making

Bias and fairness: The potential for bias in AI algorithms poses a critical ethical challenge. When trained on biased data, these algorithms can perpetuate and amplify existing biases. This is particularly relevant in healthcare decision support systems, where biased recommendations could lead to disparities in patient care. Implementing robust measures for identifying and mitigating biases within AI algorithms is imperative. This involves continuous monitoring, thorough audits, and the development of strategies to ensure that decision support systems provide fair and equitable recommendations to all patients, irrespective of demographic or other factors [17].

Transparency and explainability: The opacity of specific AI algorithms can present challenges in understanding the decision-making processes. To foster trust among healthcare professionals and patients, transparency and explainability must be prioritized in AI-driven decision support systems. Clear communication of the system's rationale, the factors influencing decisions, and the underlying processes is essential. This transparency enhances accountability and enables healthcare professionals to comprehend and trust the recommendations provided by AI, fostering a collaborative relationship between human expertise and artificial intelligence [18].

Patient privacy and data security: Safeguarding patient confidentiality is paramount, particularly as AI systems rely heavily on patient data. Ethical decision-making within AI systems necessitates implementing robust privacy measures, secure data storage practices, and strict adherence to regulatory frameworks. It is imperative to protect patient data from unauthorized access and breaches. Safeguarding patient confidentiality fulfils legal obligations and upholds a fundamental ethical responsibility that underpins patients' trust in the healthcare system [19].

Informed consent: Informed consent is foundational to ethical healthcare practices, particularly in utilizing AI within decision support systems. Patients must receive comprehensive information regarding the integration of AI into their healthcare, especially in complex situations. This includes understanding the nature of AI's role, the types of data it analyzes, and the potential implications for their care. Offering patients the opportunity to provide or withhold consent ensures their autonomy is respected, fostering trust and upholding ethical standards in patient care [20].

Human oversight: While AI can augment decision-making processes, maintaining human oversight is critical. Healthcare professionals should retain the ability to validate AI recommendations, question outcomes, and intervene when necessary. AI should be viewed as a supportive tool that complements human judgment rather than replacing it. This human-AI collaboration ensures that the ethical considerations, contextual nuances, and empathy inherent to human decision-making remain integral components of patient care, thereby striking a balance between technological advancement and human-centric healthcare [21].

Challenges and considerations

Data Privacy and Security

Patient confidentiality: Safeguarding patient privacy is a paramount concern in integrating AI within hospital management. The implementation of robust measures is essential to ensure the confidentiality of patient information. This involves employing state-of-the-art data encryption techniques to protect sensitive data from unauthorized access. Additionally, stringent access controls must be in place to limit and monitor who has permission to view or interact with patient data. Compliance with privacy regulations, such as the Health Insurance Portability and Accountability Act (HIPAA) in the United States, is crucial. By adhering to these regulations, hospitals demonstrate their commitment to maintaining patient confidentiality and fostering a foundation of trust between healthcare providers and patients [22].

Data breach risks: The escalating reliance on AI amplifies the volume of sensitive healthcare data being processed, thereby increasing the vulnerability to data breaches. Hospitals must prioritize and implement comprehensive cybersecurity measures to effectively mitigate the risks associated with unauthorized access and data breaches. This includes deploying robust intrusion detection systems, regularly updating security protocols, and conducting thorough audits of the cybersecurity infrastructure. Hospitals should also foster a culture of awareness among staff regarding potential cybersecurity threats, emphasizing the importance of vigilance and adherence to best practices. By addressing data breach risks head-on, healthcare institutions not only protect the integrity of patient information but also fortify the overall resilience of the healthcare system against evolving cyber threats [23].

Integration with Existing Systems

Interoperability: The interoperability challenge arises as many healthcare institutions operate diverse and occasionally incompatible systems. Integrating AI seamlessly into existing technologies requires a concerted effort to establish standardized interfaces and protocols. This ensures smooth communication and data exchange between disparate platforms, allowing for the cohesive integration of AI across the healthcare ecosystem. By fostering interoperability, healthcare institutions can unlock the full potential of AI applications, facilitating the exchange of critical information and promoting a more cohesive and efficient healthcare infrastructure [24].

Legacy system compatibility: The prevalence of legacy systems in hospitals poses a unique challenge to integrating AI, as these systems may not readily accommodate the technological advancements AI brings. Overcoming compatibility issues becomes imperative, and a phased approach to integration is essential to prevent disruptions in healthcare operations. This involves meticulously evaluating existing systems, identifying points of integration, and implementing strategies to adapt or upgrade legacy systems to align with AI requirements. By addressing the compatibility challenges associated with legacy systems, healthcare institutions can ensure a smooth transition to AI-driven technologies without compromising the continuity and reliability of essential healthcare services [25].

Staff Training and Adoption

Skill gaps: The effective integration of AI into hospital management hinges on the proficiency of healthcare professionals in utilizing these advanced technologies. Recognizing and addressing skill gaps becomes imperative to ensure that staff can seamlessly and effectively leverage AI tools in their daily workflows. Implementing comprehensive training programs encompassing theoretical understanding and hands-on practical experience is essential. By empowering healthcare professionals with the necessary skills, hospitals can optimize the utilization of AI, enhancing the overall efficiency of operations and ensuring that the transformative potential of these technologies is fully realized in patient care [26].

Change management: Introducing AI-driven solutions creates a fundamental shift in established workflows, necessitating a cultural transformation within healthcare organizations. Effective change management strategies are critical in this context, serving as a bridge between the existing practices and the innovative landscape introduced by AI. Gaining staff buy-in, fostering a positive attitude toward technological advancements, and minimizing resistance to change is paramount. This involves clear communication about the benefits of AI, providing ongoing support during the transition, and encouraging a culture that embraces continuous learning and adaptation. Successful change management ensures that the integration of AI is not perceived as a disruption but rather as an evolution that enhances the capabilities and effectiveness of healthcare professionals in delivering optimal patient care [27].

Ethical and Legal Implications

Liability and accountability: The integration of AI in healthcare introduces a complex ethical and legal landscape, particularly when determining responsibility in the event of errors or adverse outcomes. The intricate nature of AI-driven decision-making necessitates the establishment of clear guidelines for liability and accountability. Healthcare institutions must navigate these challenges by defining the roles and responsibilities of human operators and AI systems, ensuring transparency in decision-making processes. Establishing ethical frameworks and legal precedents that govern liability in AI-related incidents is essential for fostering trust among healthcare professionals, patients, and stakeholders. By addressing these considerations proactively, the healthcare industry can balance embracing innovation and upholding accountability in patient care [28].

Informed consent challenges: The complexity of AI-driven procedures and interventions poses challenges in obtaining patient-informed consent. Transparent communication and disclosure regarding the use of AI and its potential implications are paramount. Healthcare providers must convey information clearly and understandably, outlining the role of AI in the decision-making process, its potential benefits, and any associated risks. Ensuring that patients are adequately informed empowers them to make informed choices about their healthcare. As AI continues to evolve, healthcare institutions must remain committed to ethical communication practices, actively engaging patients in discussions about the integration of AI and respecting their autonomy in the decision-making process. This approach fosters trust and aligns with ethical principles underpinning patient-centered care [29].

Cost and Return on Investment

Initial investment vs long-term gains: The integration of AI into hospital management necessitates a significant upfront investment covering technology acquisition, staff training, and infrastructure development. Ensuring ethical and comprehensive evaluation processes are in place is crucial to ascertaining AI adoption's effectiveness and ethical implications. Evaluations must extend beyond mere financial considerations to encompass improvements in patient outcomes, operational efficiency, and overall quality of care. Demonstrating tangible and intangible benefits over the long term is imperative for garnering support, ensuring sustainability, and establishing the ethical viability of AI in shaping the future of hospital management [30].

Resource allocation challenges: Balancing the financial investment in AI with other competing priorities for resource allocation within healthcare organizations poses a significant challenge. Hospitals must navigate the delicate task of evaluating the cost-effectiveness of AI implementations while considering the broader strategic goals and financial constraints. This involves a comprehensive assessment of the potential impact of AI on operational efficiency, patient care, and overall healthcare outcomes. Strategic prioritization ensures that AI projects align with the organization's mission, values, and long-term objectives. By making informed decisions about resource allocation, hospitals can effectively integrate AI into their management practices while optimizing available resources [31].

Case studies

Successful Implementation Stories

Cleveland clinic, Ohio: The Cleveland Clinic, in a strategic collaboration with IBM, has leveraged AI to revolutionize the personalization of healthcare plans for individual patients. This innovative implementation allows the clinic to aggregate and analyze extensive datasets, enabling the tailoring of healthcare plans to each patient's unique needs and characteristics. The application of AI in healthcare planning goes beyond traditional one-size-fits-all approaches, facilitating a more nuanced and targeted strategy for patient care. By harnessing AI-driven insights, the Cleveland Clinic aims to optimize treatment plans, enhance diagnostic precision, and ultimately elevate patient outcomes. This collaborative effort exemplifies the transformative impact of AI in shaping a more patient-centric, data-driven, and personalized approach to healthcare [32].

Ciox Health, Georgia: Ciox Health, based in Georgia, has embraced AI for health data and workflow management, specifically focusing on using machine learning to enhance health information management and facilitate the exchange of health data. The integration of AI in this context has streamlined the processes of accessing clinical data, thereby improving the accuracy and flow of health information within the healthcare ecosystem. Ciox Health aims to optimize information management through machine learning algorithms, ensuring healthcare professionals have timely access to relevant data for informed decision-making. This application underscores the transformative potential of AI in optimizing healthcare workflows and enhancing the efficiency of health data exchange, contributing to improved patient care and outcomes [32].

Johns hopkins hospital, Maryland: Johns Hopkins Hospital, situated in Maryland, has partnered with GE to implement predictive AI techniques to enhance the efficiency of hospital visits. This strategic implementation has resulted in faster hospital visits, courtesy of AI-driven predictive analytics. By leveraging these techniques, Johns Hopkins Hospital seeks to optimize resource allocation, reduce wait times, and enhance the overall efficiency of hospital operations. The integration of predictive AI addresses operational challenges and directly contributes to an improved patient experience by minimizing delays and streamlining the entire hospital visitation process. This collaboration serves as a noteworthy example of how AI applications can lead to tangible improvements in healthcare operations and patient satisfaction [32].

These success stories demonstrate the diverse applications of AI in healthcare, ranging from personalized healthcare plans to workflow management and predictive techniques for improved efficiency. The acceptance and successful integration of AI in these hospitals are valuable examples for the broader healthcare industry [32].

Lessons Learned from Failures

Data quality and understanding the problem: Many AI projects fail due to issues related to data quality, data labeling, and a need to understand of the problem being addressed. It is essential to spend time framing the right questions, exploring more data, and testing the understanding of the problem to ensure the success of AI projects [33].

Transparency and ethical concerns: Failures underscore the significance of transparency, interpretability, and continuous monitoring to ensure that AI models behave as intended and deliver real value. Addressing ethical concerns and avoiding overreliance on black-box models is crucial for the success of AI projects [34].

Strategic direction and thorough monitoring: The importance of strategic direction, thorough monitoring, and evaluation is highlighted to ensure AI's safe and effective implementation in healthcare. Clear communication and coordination are essential for increasing public confidence in adopting new AI technologies [35].

Continuous learning and adaptation: Embracing a mindset of continuous learning and adaptation is crucial for the AI community to harness the immense potential of AI for the betterment of society. Analyzing failures critically and constructively can help identify and avoid common mistakes and challenges in AI project management [34,36].

These lessons from AI project failures serve as valuable insights for organizations and professionals looking to navigate the complexities of implementing AI in healthcare and other domains. By learning from these failures, it is possible to refine AI projects, address challenges, and maximize the potential benefits of AI technologies.

Impact on Patient Outcomes

Integrating artificial intelligence (AI) in healthcare has significantly improved patient outcomes. AI has enabled more accurate diagnoses, personalized treatment plans, and improved health outcomes [37]. By analyzing vast quantities of clinical data, AI enables medical professionals to identify disease markers and trends quickly and accurately, leading to better care outcomes and reduced healthcare costs [37]. AI tools can also assess treatments' effectiveness and suggest real-time adjustments, improving clinical outcomes [37]. Additionally, AI can automate mundane administrative tasks, freeing up time for medical professionals to focus on direct patient care and ultimately enhancing the overall patient experience [37,38]. However, there are concerns about the safety and reliability of AI systems, which could cause errors and lead to adverse effects [39]. Addressing these concerns and ensuring transparency and accountability are crucial for successfully implementing AI in healthcare and improving patient outcomes.

Future trends and emerging technologies

Advances in AI for Healthcare

Explainable AI (XAI): Future AI systems in healthcare are anticipated to prioritize explainability, a key feature to enhance the understanding and trustworthiness of AI algorithms among healthcare professionals. Explainable AI (XAI) addresses the inherent complexity of advanced models, making them more transparent and interpretable. By providing insights into the decision-making processes of AI algorithms, XAI facilitates collaboration between AI systems and human practitioners. This transparency is crucial in healthcare settings where clear comprehension of AI-generated recommendations is paramount for making informed clinical decisions. As the healthcare industry embraces AI, the emphasis on explainability contributes to harmonizing technology into existing practices, ensuring a seamless partnership between AI and human expertise [40].

Federated learning: Federated learning emerges as a pivotal advancement with profound implications for preserving data privacy in healthcare. This approach enables machine learning models to be trained collaboratively across decentralized devices or servers without exchanging raw data. In healthcare settings, federated learning holds the potential to facilitate collaborative model training across multiple hospitals, research institutions, or healthcare providers. By allowing the aggregation of insights without compromising patient privacy, federated learning addresses the sensitive nature of healthcare data. This innovative approach enhances data security and promotes collaboration in large-scale healthcare research endeavors, laying the foundation for more robust and privacy-preserving AI applications [41].

Predictive genomics: Integrating AI with genomics data marks a significant advancement with transformative implications for personalized medicine. When applied to genetic information, AI algorithms can predict disease risks, identify optimal treatment strategies, and contribute to developing targeted therapies. This intersection of AI and genomics data has the potential to revolutionize the field of precision medicine, tailoring medical interventions to an individual's genetic makeup. By leveraging predictive genomics, healthcare professionals can usher in an era of more personalized and effective treatment plans, optimizing patient outcomes and contributing to advancements in our understanding of genetic influences on health and disease [42].

Integration of AI with Emerging Technologies

Blockchain for data security: Integrating blockchain technology with AI in healthcare presents a powerful solution for enhancing data security and integrity. Blockchain's decentralized and tamper-resistant nature establishes a secure and transparent framework for managing healthcare data. By utilizing a distributed ledger, blockchain reduces the risk of unauthorized access, data breaches, or tampering. The immutability of blockchain records ensures the integrity of health data, providing a robust foundation for AI applications in healthcare. This integration safeguards sensitive information and fosters trust among patients and healthcare professionals in securing their health records [43].

Internet of things (IoT) and edge computing: The convergence of AI with the Internet of Things (IoT) and edge computing can revolutionize real-time data processing in healthcare. Connected medical devices, wearables, and sensors generate vast amounts of data, and AI algorithms at the edge can analyze this data in real-time. This facilitates quicker and more responsive healthcare interventions, allowing for timely insights and decision-making. The synergy of AI with IoT and edge computing enhances the efficiency of data analysis and enables healthcare professionals to deliver personalized and proactive care, leveraging insights from real-time data generated by a network of interconnected devices [44].

5G technology: The widespread adoption of 5G technology represents a significant leap forward in healthcare capabilities. With faster and more reliable data transmission, 5G technology enhances access to medical data, particularly for resource-intensive applications like AI. This advancement translates to improved telemedicine capabilities, enabling seamless, high-quality virtual healthcare experiences. In addition, 5G's low latency and high bandwidth are particularly advantageous for AI-driven applications that require real-time processing, such as remote patient monitoring and augmented reality in surgical procedures. Integrating 5G technology in healthcare infrastructure accelerates data transfer and unlocks new possibilities for delivering advanced, connected healthcare services [45].

Potential Disruptions in Hospital Management

Decentralized healthcare models: The evolving landscape of telemedicine, remote patient monitoring, and AI-driven diagnostics is paving the way for decentralized healthcare models. This paradigm shift challenges the traditional hospital-centric approach, offering the potential for more personalized and accessible healthcare services delivered beyond the confines of traditional hospital settings. Advances in technology enable healthcare to extend into the community, empowering patients with greater control over their health. Decentralized models may leverage telehealth platforms, wearables, and AI to enhance preventive care, diagnostics, and ongoing management of health conditions, ultimately fostering a more patient-centric and flexible healthcare ecosystem [46].

Human-machine collaboration: The future envisions an increased collaboration between healthcare professionals and AI systems, where AI acts as a supportive tool in decision-making, diagnostics, and treatment planning. Striking the right balance between human expertise and AI capabilities will be crucial for optimizing patient care. AI can assist in processing vast amounts of data, identifying patterns, and providing evidence-based recommendations, allowing healthcare professionals to focus on the nuanced aspects of patient care that require empathy, emotional intelligence, and contextual understanding. This collaborative approach aims to harness the strengths of both human and machine intelligence, ensuring a holistic and patient-centred healthcare experience [47].

Digital therapeutics: The emergence of digital therapeutics, leveraging AI to deliver personalized, software-based interventions, can disrupt traditional treatment approaches. AI-driven digital therapies extend beyond traditional pharmaceutical interventions, offering targeted and adaptive solutions for managing chronic conditions, mental health issues, and rehabilitation. These therapies can be tailored to individual patient needs, continuously adapting based on real-time data and patient responses. To complement traditional healthcare approaches, digital therapeutics represent a new frontier in healthcare delivery, providing scalable, accessible, and potentially cost-effective solutions for various medical conditions. Integrating AI in digital therapeutics opens avenues for innovative and personalized interventions, transforming the landscape of patient care [48].

## Conclusions

AI's transformative impact on hospital management presents opportunities and challenges. Integrating AI across administrative, clinical, and patient engagement domains can enhance efficiency and revolutionize patient care. However, navigating ethical, legal, and operational complexities is vital. Balancing innovation with ethics, prioritizing data security, and addressing staff training needs are critical. To succeed, we must invest in education, uphold ethical standards, foster collaboration, advocate for sensible policies, and embrace innovation. By doing so, we can unlock AI's potential, offering more effective and accessible patient care in hospital management.
